# Randolph M. Nesse, *Good Reasons for Bad Feelings: Insights from the Frontier of Evolutionary Psychiatry*

**DOI:** 10.1093/emph/eoaa002

**Published:** 2020-01-24

**Authors:** Steven Austad

**Affiliations:** Department of Biology University of Alabama at Birmingham CH 464, 1720 2nd Avenue South, Birmingham, AL 35294-2172, USA

The modern world, it seems, needs all the psychiatric help it can muster. In Europe, 38% of the population suffers from at least one mental disorder annually [1]. This is similar to the 35% of global college students who report having a mental disorder [2]. In America, >70 000 people died from drug overdoses in 2017 [3]—the latest year for which reliable statistics are available—and nearly 50 000 more died from suicide [4]. These numbers do not include an estimated 88 000 deaths from alcohol abuse or nearly 500 000 more attributed to cigarette smoking. Against all evolutionary logic, we seem to be working hard to do ourselves in. 

Against this backdrop of disturbing news, I picked up Randy Nesse’s engaging new book, *Good Reasons for Bad Feelings: Insights from the Frontier of Evolutionary Psychiatry* (Dutton, $28.00), in the hope that it would help me understand a bit more about the human behavior and especially mental states, disorders, and diseases than I currently did. I was not disappointed.

Nesse, of course, is one of the founders of the field of evolutionary medicine. His 1991 *Quarterly Review of Biology* paper with George Williams brought together many of the ideas that still drive the field. Surprisingly, although I have read many of his papers since then, and heard him give more than a few talks, I had never before come to know Nesse’s outlook on his own chosen discipline, psychiatry.

The book is gracefully written for an educated lay audience in four parts. Part 1 (*Why Are Mental Disorders So Confusing?*) lays out the basics of an evolutionary thinking about mental disorders and also traces Nesse’s own gradual awakening to what an evolutionary perspective could add to his understanding of his own patients, even though it might not necessarily suggest new therapies. Nesse is a traditionally trained, and before he encountered evolutionary thinking, a traditionally trained practicing psychiatrist. Consequently, one thing this book is not is an attack on traditional psychiatry. Given his long history of listening carefully to his patients, Nesse is aware that traditional, talking and drug-treatment based, psychiatric treatment is often helpful and necessary. What an evolutionary perspective has to offer is to bring some order to a chaos of symptoms and overlapping diagnoses as well as insight into the origins of moods, emotions and psychiatric diseases.

The main messages I took from this part of the book are first that psychiatry too often fails to distinguish symptoms from diseases. This can lead to ignoring the importance of the situations that arouse extremes of emotion, such as anxiety and depression that psychiatrists deal with on a daily basis. Second, Nesse emphasizes a core feature of evolutionary medicine, namely that there are good evolutionary reasons, such as the mismatch between the modern environment and the environment in which we evolved, that natural selection has left us vulnerable to certain diseases, including mental diseases. Third, he points out a core problem for psychiatric diagnosis is the lack of a coherent perspective on the normal useful functions of moods and emotions. His book, in fact, provides such a perspective.

The second part of the book (*Reasons for Feelings*) elaborates on this third point. Even if you are not a particularly introspective person—and I am not—this part of the book will make you freshly evaluate your own moods and emotions. Nesse’s hypothesis is that even seemingly negative emotions such as anxiety, depression, anger and guilt exist because they are part of a naturally selected system of adaptive responses to specific situations and that absence of such responses can be harmful. Painful emotions, for instance, can motivate us to change, escape, or avoid certain situations. These are the good reasons for bad feelings—his book’s title. Invoking what he calls the Smoke Detector Principle, the idea that some extreme emotions can be thought of as low-cost warnings about potentially life-threatening situations. Panic, for instance, might be useful if it increased our running speed when threatened by a lion. However, like any sensitive danger detector, sometimes it will issue false alarms. Panic disorder, for instance, he interprets as a false alarm in this emergency response system.

He also considers mood, that is, long-term pervasive mental states, as adaptive responses to life situations. Low mood, that is, mild depression or low-grade psychic pain, he interprets as something that may prompt us to give up unattainable goals and consider alternatives, whereas high mood, a state of energetic excitement, can facilitate progress toward life goals. When what he calls the ‘moodostat’ fails due to either genetics (for instance, bipolar disorder has a heritability of 70%), life events or both, we experience these moods inappropriately. They may become extreme and we end up in the psychiatrist’s office.

The third part of the book (*The Pleasures and Perils of Social Life*) addresses social life, examining how we interact or respond to others. Noting that we are above all a social species, he investigates how what other people think about us, or *what we think* other people think of us, has major consequences. In this part of the book, evolutionary psychology can merge in satisfying ways with more traditional psychology. For instance, Freud discovered the unconscious mind, which Nesse identifies as having adaptive value, causing people to act in ways that may have been evolutionarily beneficial without us necessarily being aware of it. Trivers’ idea that self-deception evolved to make us better able to deceive others is a good example of the adaptive unconscious. This part of the book also addresses difficult questions, such as is grief an adaptive state. One of the aspects of this book that I most appreciated is that Nesse does not pretend to understand, or be able to explain, everything. He offers hypotheses but rightly notes that they are only that. He also goes out of his way to give credit to others.

The final section addresses sexual behavior and what he calls out-of-control psychiatric behaviors. In terms of sexual behavior, he identifies a key problem of human existence. As he nicely summarizes it, ‘selection shaped our brains and bodies to maximize reproduction—at enormous costs to human happiness’. The section also addresses addiction and has possibly the most sensible explanation for the pervasiveness of drug addiction I have encountered. It goes like this. Humans (and other animals) discovered accidentally that certain natural products can hijack our pleasure system and elicit euphoria. Over the course of our evolutionary history, however, these products were rare and unavailable in high concentration. Consequently, evolution never gave us defenses against overindulgence. However, modern science has made these products available in pure, high concentrations where the euphoria can be almost instantaneous. Is it any wonder, that overindulgence has become so pervasive?

Given my own interests in aging and sex differences, there are topics not covered about which I would have loved to know Nesse’s thoughts. I am sure he would have something interesting to say, for instance, about why psychiatric diseases arise most often early in life rather than late in life like virtually all chronic physical diseases. Also, why are there such dramatic sex differences in depression and autism?

In summarizing a few—and only a few—topics covered in this book, I have not done justice to its breadth nor to the history of psychiatric thought nor the various fascinating case histories that Nesse describes. Suffice it to say that there is scarcely a page that will not make the reader pause for thought and probably for self-examination.

How would this book work in an educational context? As someone whose book on aging has been used pedagogically although that was not my original intent, I have given a bit of thought to what makes for an effective classroom text. There is the content, of course, and this book has more than enough engaging content to be useful in any psychology course. Its broad concepts could also be useful to students studying neuroscience, environmental health, possibly even some social science courses. It has an excellent index and several useful explanatory figures and charts. Other features that would make it even more useful in the classroom—something to be considered for future editions, perhaps—might include chapter summaries, text boxes with key ideas or case histories and photographs with short biographic summaries of key figures mentioned in the text.
